# Graph‐guided frequency‐enhanced state space network for 3D spine segmentation from MR images

**DOI:** 10.1002/acm2.70481

**Published:** 2026-02-12

**Authors:** Linghui Hong, Zhengchao Zhou, Wanbo Xu, Pingping Wang, Benzheng Wei

**Affiliations:** ^1^ Center for Medical Artificial Intelligence Shandong University of Traditional Chinese Medicine Qingdao China; ^2^ Qingdao Academy of Chinese Medical Sciences Shandong University of Traditional Chinese Medicine Qingdao China; ^3^ Qingdao Key Laboratory of Artificial Intelligence Technology in Traditional Chinese Medicine Qingdao China; ^4^ Department of Medical Imaging Center Qilu Hospital of Shandong University Dezhou Hospital Dezhou China; ^5^ Dezhou Key Laboratory of Intelligent Imaging Dezhou China

**Keywords:** frequency dynamic convolution, graph convolutional network, spinal MRI segmentation, state space model

## Abstract

**Background:**

Accurate spinal MRI segmentation is essential for computer‐aided diagnosis of spinal diseases. Existing methods have limitations in global semantic modeling and boundary delineation due to complex anatomy and imaging artifacts.

**Purpose:**

Our work aimed to propose a novel Graph‐Guided Frequency‐Enhanced State Space Network (GF‐SSNet) method to achieve more accurate 3D multi‐modal spine MRI automatic segmentation, addressing the limitations of existing algorithms in global semantic modeling of high‐dimensional voxel space, cross‐modal information synergistic perception, and fine boundary identification of anatomically similar tissues, thereby providing technical support for intelligent diagnosis and precision medicine of spinal diseases.

**Methods:**

The proposed network is based on the GF‐SSNet architecture. During encoding, a dual frequency‐spatial feature enhancement mechanism is employed, which adaptively fuses local frequency dynamic features and global spatial long‐range dependencies through Frequency Dynamic Convolution (FDConv) and Three‐Directional Mamba‐based state space model (TD‐Mamba). At the bottleneck, Position‐Aware Attention Fusion (PAAF) and Graph Convolutional Networks (GCN) are integrated to explicitly encode topological anatomical constraints between vertebrae, enhancing the global perception capability of spinal continuity structures. During decoding, a Depth‐aware Progressive Upsampling (DAPU) strategy is introduced to effectively alleviate the reconstruction loss of fine‐grained spatial information. The entire framework achieves end‐to‐end automatic segmentation of multi‐modal MR images.

**Results:**

On the normal test set, GF‐SSNet outperformed all baselines across all metrics. Specifically, Dice and IoU Means reached 92.04 ± 0.06% and 85.29 ± 0.10%, exceeding the best baseline results of 89.81 ± 0.61% and 81.51 ± 0.91%. HD95 and ASSD were significantly reduced to 3.06 ± 0.46 mm and 0.612 ± 0.018 mm, compared to top‐tier baseline values of 4.76 ± 1.09 mm and 1.14 ± 0.01 mm, respectively. On an independent pathological test set with various spinal pathologies, GF‐SSNet maintained superior performance with Dice Mean of 87.60 ± 0.10%, still outperforming all baseline methods. The 4.4 percentage point performance decline from normal cases primarily stemmed from intervertebral disc segmentation challenges in degenerative conditions, while vertebrae segmentation remained robust. Ablation studies confirmed significant contributions of all proposed components. The proposed HFD‐Tversky loss outperformed conventional losses. All performance differences were statistically significant after correction for multiple comparisons.

**Conclusion:**

GF‐SSNet demonstrates performance in spinal segmentation through adaptive fusion of frequency features and global dependencies, providing technical support for intelligent spinal disease diagnosis.

## 1 INTRODUCTION

Spinal diseases, as a global chronic degenerative pathological condition, have become a major public health challenge.^1^, ^2^ According to World Health Organization statistics, approximately 80% of the population experiences at least one episode of spine‐related pain during their lifetime,^3^ and chronic lumbago has become one of the leading causes of disability worldwide.^4^ Spinal magnetic resonance imaging (MRI), with its advantages of no radiation exposure and excellent soft tissue contrast, has been established as the gold standard imaging method for spinal disease diagnosis.^5^ The comprehensive utilization of multi‐sequence MRI can provide more comprehensive pathophysiological information, significantly improving diagnostic accuracy and precision of treatment decision‐making.^6^ Therefore, precise segmentation of various tissue structures in spinal MRI has important clinical significance for disease‐assisted diagnosis, surgical planning, and treatment evaluation.^7^

Deep learning models have achieved significant progress in spinal segmentation. CNN‐based methods, such as U‐Net^8^ and nnU‐Net,^9^ demonstrate excellent segmentation performance through automatic feature extraction via convolutional operations and encoder‐decoder architectures. These methods leverage skip connections and multi‐scale feature fusion to capture both local details and global context. Advanced variants like U‐Net++^10^ and U‐Net 3+^11^ further enhance performance through dense skip connections and full‐scale feature aggregation, enabling more precise localization of spinal structures. Transformer‐based methods, such as TransUNet,^12^ UNETR,^13^ and Swin‐UNETR,^14^ achieve precise segmentation through self‐attention mechanisms that capture long‐range dependencies and global contextual information. These approaches excel at modeling relationships between distant anatomical structures, which is particularly beneficial for understanding the continuous nature of spinal anatomy. Recent work like SymTC^15^ demonstrates the potential of hybrid CNN‐Transformer architectures for lumbar spine segmentation tasks.

Although CNN‐based and Transformer‐based methods have achieved significant progress in medical image segmentation, existing methods still face key challenges when processing the complex anatomical structure of the spine. Traditional CNNs are limited by local receptive fields, making it difficult to model the long‐range dependencies essential for spinal anatomy.^9^ While Transformer‐based methods offer strong global modeling capabilities, they struggle to capture fine‐grained local features.^12^ Moreover, their quadratic computational complexity poses challenges for high‐resolution 3D medical images.

Recently, structured State Space Models (SSMs) have demonstrated efficiency and effectiveness in long sequence modeling,^16^ with the latest research further proving Mamba's superior performance in long sequence data analysis tasks.^17^ SSMs like Mamba offer linear computational complexity while maintaining the ability to capture long‐range dependencies, making them particularly suitable for processing high‐dimensional medical data. Recent applications in medical imaging, such as U‐Mamba^18^ and SegMamba,^19^ have shown promising results for biomedical image segmentation tasks. However, the scanning operation (Scan) adopted by Mamba essentially compresses high‐dimensional data into one‐dimensional sequences for processing. Its state update rules still have limitations in capturing the geometric structural features inherent in 3D spinal spaces, and the related modeling capabilities urgently need further exploration and improvement.

Despite the continuous emergence of various segmentation models for 3D spinal images in recent years,^20^ achieving significant progress in both accuracy and efficiency, several key aspects remain neglected in research. Most prominently overlooked is the full utilization of frequency domain information: spinal images carry distinctly different structural and semantic information across different frequency components.^21^ High‐frequency regions often correspond to fine anatomical boundaries, tissue interfaces, and pathological changes, while low‐frequency components contain global structural patterns, organ shapes, and overall anatomical organization.^22^ However, existing methods mostly focus only on spatial domain modeling and fail to establish effective frequency domain perception mechanisms.^23^, ^24^

Recent work in frequency domain medical image segmentation, such as the Frequency Selection Segmentation Network (FSSN)^25^ and Spatial‐Frequency Dual‐Domain Attention Network (SF‐UNet),^26^ has demonstrated that incorporating frequency domain processing can significantly improve segmentation performance. These approaches show that frequency domain features provide complementary information to spatial domain features, particularly for capturing both local details and global context simultaneously. On the other hand, the spine itself has clear and strict anatomical prior structures, such as the continuous arrangement of vertebrae, topological consistency between vertebral structures, and predictable spatial relationships. However, current models have not systematically and deeply integrated such morphological knowledge into the segmentation process. This represents a significant opportunity for improvement, as anatomical priors could guide the learning process and improve segmentation accuracy, particularly in challenging cases with pathological variations or imaging artifacts. These limitations constrain the performance of existing methods in complex spinal segmentation tasks.

To address these challenges, this paper introduces a Graph‐guided Frequency‐enhanced State Space Network (GF‐SSNet) for precise 3D spinal MRI segmentation. Frequency Dynamic Convolution (FDConv) dynamically captures anatomical features across frequency domains through adaptive weight selection and frequency band modulation. Three‐directional Mamba (TD‐Mamba), inspired by Mamba's capability of modeling long‐range dependencies with linear complexity, extends this approach to three‐dimensional space by modeling long‐range dependencies along depth, height, and width dimensions, ensuring simultaneous capture of axial, coronal, and sagittal anatomical structural information. This addresses the limitations of traditional Mamba in processing 3D volumetric medical data while achieving deep integration of frequency and state space representations. At the bottleneck layer, a Position‐Aware Attention Fusion (PAAF) module enables position‐sensitive cross‐modal feature integration across sequences. Inspired by the fact that Graph Convolutional Networks (GCNs) offer a promising alternative by explicitly modeling vertebral relationships as graph structures and naturally capturing both local morphology and global spinal topology while maintaining computational efficiency, we employ GCNs to explicitly model the topological relationships between vertebrae.^27^ The decoder incorporates a Depth‐Aware Progressive Upsampling (DAPU) module that learns slice‐specific importance through depth attention and employs boundary enhancement strategies to preserve anatomical structural details, ensuring high‐resolution segmentation output. During the training process, we employ a novel Hybrid Frequency‐Domain Aware Tversky Loss (HFD‐Tversky) function to enhance segmentation accuracy by addressing class imbalance between vertebrae and intervertebral discs while strengthening boundary identification through frequency‐domain gradient information. This architecture effectively addresses critical challenges in 3D spinal segmentation, including long‐range dependency modeling, multi‐modal feature fusion, and boundary refinement. Comprehensive experimental validation confirms the superior performance of the proposed approach. The main contributions of this work include:

1. We proposed GF‐SSNet, a novel architecture that addresses key challenges in 3D spinal MRI segmentation by seamlessly integrating three complementary components: frequency‐domain dynamic convolution for multi‐frequency feature extraction, graph convolutional networks for structural topology modeling, and state space models for long‐range dependency modeling. This integration effectively addresses the neglect of synergistic processing of complex topological relationships, frequency‐domain information, and local anatomical details in existing studies, thereby achieving more accurate and robust segmentation.
2. We designed a suite of specialized feature processing modules: a spatial enhancement encoding module, a position‐aware attention fusion module for multi‐modality integration, a graph‐guided feature enhancement module, and a depth‐aware progressive upsampling module. These modules enable comprehensive multi‐scale feature extraction and fusion, substantially enhancing segmentation performance.
3. We introduced a Hybrid Frequency Domain‐aware Tversky (HFD‐Tversky) loss function that incorporates differential weighting for vertebrae and intervertebral disc segmentation targets while integrating frequency domain gradient constraints. This approach effectively addresses class imbalance and boundary delineation challenges in spinal segmentation, substantially improving segmentation accuracy for small‐volume intervertebral discs and vertebral boundary precision.
4. Comprehensive experiments and ablation studies on multi‐modal MRI datasets demonstrated that GF‐SSNet outperforms existing methods across key evaluation metrics, including Dice coefficient and Hausdorff distance, confirming the effectiveness of each proposed component.

## 2 METHODS

### 2.1 Dataset definition

In this work, the dataset used in this study consists of 190 sets of clinical lumbar sagittal MR images collected from Qilu Hospital of Shandong University Dezhou Hospital. Each set includes both T1‐weighted images and T2‐weighted images, with a slice thickness of 4.0 mm, an inter‐slice spacing of 0.5 mm, 11 slices per sequence, and image sizes ranging from 408 × 489 to 512 × 384 pixels. The paired T1 and T2‐weighted images were acquired sequentially within the same scanning session with identical imaging parameters, ensuring inherent spatial alignment (verified mean alignment error: < 1 mm translation, < 1° rotation). All images were annotated by two senior radiologists (with 15 and 12 years of musculoskeletal imaging experience, respectively) using a consensus protocol. The initial segmentation was performed using ITK‐SNAP software with simultaneous consultation of both T1 and T2‐weighted images to ensure optimal boundary determination. Segmentation masks were created in T2 image space while cross‐referencing T1 images for anatomical verification. All annotations were independently reviewed by the second radiologist, and discrepancies were resolved through consensus discussion, clearly delineating the relative positions of vertebral bodies and intervertebral discs.

The 190 sets of images were randomly divided into training, validation, and test datasets, comprising 126 sets for training, 32 sets for validation, and 32 sets for testing, respectively. This division ensures sufficient data for model training while maintaining independent datasets for hyperparameter tuning and final performance evaluation. The validation set was used exclusively for hyperparameter tuning and model selection, while the test set was held out and used only for final performance evaluation to ensure unbiased assessment.

To evaluate the robustness and clinical applicability of our method on various spinal pathologies, we constructed an additional independent pathological test set consisting of 16 lumbar spine MRI cases from patients diagnosed with common degenerative spinal diseases, including disc degeneration, disc herniation, and spinal stenosis. These cases were collected from Qilu Hospital of Shandong University Dezhou Hospital under the same imaging protocol and annotation standards as the original dataset. This pathological test set is completely independent from the training, validation, and original test sets, providing a rigorous evaluation of model performance on clinically relevant pathological conditions commonly encountered in clinical practice.

During the preprocessing stage, all images were standardized by setting the number of slices to 48 and resizing to 256 × 256 pixels. Data augmentation techniques, including random cropping and random rotation, were applied to improve the model's generalization capability.

This study and all research are approved and conducted following relevant guidelines and regulations.

### 2.2 Framework overview

**FIGURE 1:**
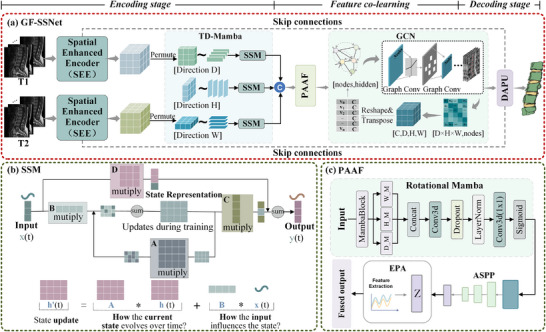
The overall architecture of GF‐SSNet. (a) Pipeline of GF‐SSNet for 3D spinal MR image segmentation with dual‐branch encoding, tri‐directional mamba (TD‐Mamba) processing, position‐aware attention fusion, GCN‐based feature co‐learning, and DAPU decoding; (b) state space model (SSM) showing matrix‐based state evolution mechanism; (c) position‐aware attention fusion (PAAF) module with multi‐directional processing and multi‐scale feature extraction.

This paper proposed GF‐SSNet for lumbar spine magnetic resonance 3D image segmentation. The architecture is illustrated in Figure 1(a). In the encoding stage, T1 and T2 dual‐modal images are input, and features are extracted through Spatial Enhancement Encoders (SEE), respectively. SEE combines the advantages of Frequency Dynamic Convolution (FDConv) and State Space Models, simultaneously capturing local texture features and long‐range spatial dependencies. The feature fusion stage deeply fuses the dual‐modal features. Following this, the PAAF module is applied to further enhance feature representation. Specifically, Graph Convolutional Networks (GCN) are utilized to transform the encoded features into graph structures, incorporating anatomical prior knowledge into the model. The decoding stage employs Depth‐Aware Progressive Upsampling (DAPU) modules, progressively restoring the spatial resolution of feature maps through four upsampling operations. Skip connections in the overall architecture ensure effective transmission of high‐resolution features and alleviate the vanishing gradient problem during deep network training.

#### 2.2.1 Spatial enhancement encoder (SEE)

Lumbar spine structures exhibit significant morphological variations among different patients, making it difficult for fixed convolutional kernels to adapt to such diversity; lumbar spine structures span multiple scanning planes, requiring the establishment of long‐range spatial dependencies across slices; the contrast between vertebral boundaries and surrounding soft tissues is relatively low, making local feature extraction susceptible to noise interference. SEE is proposed to address these issues.

**FIGURE 2:**
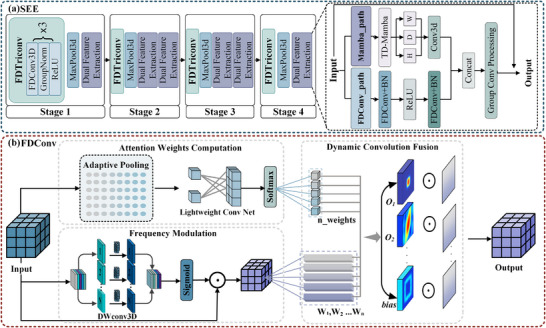
The detailed architectures of key modules in GF‐SSNet. (a) Spatial Enhanced Encoder (SEE) module with four progressive stages, integrating dual‐path processing of Frequency Dynamic Convolution (FDConv) and TD‐Mamba for hierarchical feature extraction; (b) Frequency Dynamic Convolution (FDConv) component with attention weights computation and dynamic convolution fusion for adaptive feature processing.

As shown in Figure 2(a), SEE adopts a four‐stage hierarchical architecture with two core components: FDConv and dual feature extraction pathways. FDConv adaptively adjusts convolutional parameters based on the frequency domain characteristics of input features, effectively capturing anatomical information across different frequency bands. The dual feature extraction pathways implement frequency domain feature extraction based on FDConv and spatial domain feature extraction based on TD‐Mamba, achieving fusion to complete an efficient multi‐modal feature representation.

(1) Frequency Dynamic Convolution (FDConv)

In spinal MRI, different frequency components encode distinct information crucial for accurate segmentation. Low‐frequency components primarily capture fundamental tissue contrast: in T1‐weighted images, fatty bone marrow within vertebral bodies exhibits high signal intensity while intervertebral discs show low intensity; in T2‐weighted images, the water‐rich nucleus pulposus presents high signal intensity whereas vertebral bodies exhibit relatively low intensity. Middle‐frequency components encode morphological features such as vertebral body contours and intervertebral disc boundaries, while high‐frequency components contain fine textural details and pathological alterations.

Different MRI sequences exhibit distinct frequency sensitivities. T1‐weighted sequences emphasize anatomical structure visualization and benefit from enhanced low‐to‐middle frequency information to improve tissue boundary delineation. In contrast, T2‐weighted sequences demonstrate higher pathological sensitivity and require enhanced middle‐to‐high frequency components to capture subtle lesion variations. The adaptive frequency modulation mechanism of FDConv is specifically designed to exploit these sequence‐specific frequency characteristics.

FDConv enhances the modeling capability for complex anatomical structures by introducing a content‐adaptive multi‐kernel mechanism and frequency‐selective modulation. As illustrated in Figure 2(b), FDConv comprises three parallel processing branches: the attention weight computation branch captures global context through global adaptive pooling and learns the weight distribution for N convolutional kernels via two 1×1×1 convolutional layers; the frequency modulation branch employs depthwise convolution to process each channel independently, generating spatially adaptive modulation coefficients that selectively enhance important frequency components while suppressing noise; the multi‐weight dynamic fusion stage maintains N independent learnable convolutional kernels {W_1_…W_n_}, which independently process the modulated features and are adaptively combined through attention weights.

The detailed operational procedure of frequency modulation is defined as follows. Given an input feature map $X\in R^{C\times D\times H\times W}$, where C denotes the number of channels, D, H, and W represent the depth, height, and width dimensions, respectively, the frequency‐modulated output $X_{\mathrm{mod}}$ is computed as:

(1) $$X_{\mathrm{mod}}=X⊙\sigma (\mathrm{Con}v_{\mathrm{dw}}(X)),$$

where X represents the input feature map, $X_{\mathrm{mod}}$ denotes the frequency‐modulated feature map, ⊙ denotes element‐wise multiplication (Hadamard product), $\mathrm{Con}v_{\mathrm{dw}}$ denotes depthwise separable convolution, with independent convolutional kernels for each channel specifically learning the frequency response characteristics of that channel, and σ(·) represents the Sigmoid function generating frequency‐selective weights in the range [0,1]. Through this element‐wise multiplication operation, the network learns to adaptively emphasize or suppress different frequency components at each spatial location, enabling frequency‐selective feature extraction. The final multi‐kernel fusion output is formulated as:

(2) $$Y=\sum_{i\;=1}^{N}\alpha_{i}\cdot (W_{i}*X_{\mathrm{mod}})+b,$$

where $\alpha_{i}$ is the attention weight corresponding to the i‐th convolutional kernel, $\left\{W_{i}\in R^{C_{\mathrm{out}}\times C_{\mathrm{in}}\times k\times k\times k}\right\}$ given learnable convolutional kernel parameters, ∗ represents the convolution operation, $b\in R^{C_{\mathrm{out}}}$ is the shared bias term.

This frequency‐adaptive mechanism enables FDConv to dynamically adjust its feature extraction strategy according to the imaging characteristics of different MRI sequences, achieving multi‐frequency collaborative modeling of spinal anatomical structures.

(2) TD‐Mamba

The state dimensions and convolutional kernel sizes of TD‐Mamba are adaptively adjusted according to feature hierarchy. The Mamba outputs from three directions are fused through feature concatenation and 1 × 1 × 1 convolution, ensuring the model simultaneously captures structural information from axial, coronal, and sagittal anatomical planes. This addresses the fundamental limitations of traditional Mamba when processing 3D volumetric medical data. Standard Mamba, originally designed for 1D sequential data, exhibits poor performance in 3D medical imaging due to several factors: loss of spatial topological information during sequence flattening, inability to simultaneously capture relationships across axial, coronal, and sagittal anatomical planes, and lack of directional awareness capability in the state transition mechanism.

As shown in Figure 1(a, b), TD‐Mamba overcomes these limitations through a comprehensive three‐directional (depth (D), height (H), and width (W)) scanning strategy. Depth‐directional processing captures inter‐vertebral continuity corresponding to sagittal plane observation, height‐directional processing models bilateral symmetry reflecting coronal plane characteristics, and width‐directional processing extracts anteroposterior relationships from axial slices.

Each directional Mamba module independently learns sequence dependencies in that dimension, with the core lying in the state space equations:

(3) $$h_{t}=Ah_{t-1}+Bx_{t},$$

(4) $$y_{t}=Ch_{t},$$

where h(t) represents the hidden state vector, x(t) is the input signal, y(t) is the output signal, A, B, and C are the state matrix, input matrix, and output matrix, respectively.

TD‐Mamba adaptively adjusts state dimensions and convolution kernel sizes according to feature hierarchy levels. The Mamba outputs from three directions are fused through feature concatenation and 1×1×1 convolution, ensuring the model simultaneously captures structural information from the axial, coronal, and sagittal anatomical planes.

#### 2.2.2 Feature co‐learning and graph convolution enhancement

Feature co‐learning achieves a deep fusion of multi‐modal features through collaborative learning mechanisms and introduces graph convolutional networks to explicitly model the topological structural relationships of the spine.

(1) Position‐aware attention fusion (PAAF)

As illustrated in Figure 1(c), a position‐aware attention fusion mechanism is designed to achieve collaborative learning of multi‐modal features. This mechanism first enhances the deep features of dual‐branch encoders through global context enhancement using Rotational Mamba, employing multi‐directional scanning strategies to generate enhanced features:

(5) $$F_{i}^{\mathrm{enh}}=\mathrm{LayerNorm}\;(F_{i}^{\mathrm{rot}})⊙\sigma (W_{g}F_{i}^{\mathrm{rot}}),$$

where $W_{g}\in R^{C\times C}$ are gating weights, $F_{i}^{\mathrm{rot}}$ was obtained through fusion after processing in three directions via Mamba.

Subsequently, the module adopts a channel‐wise decomposition strategy to construct cross‐modal feature representations, combining corresponding channel features and compressing them into single channels through 1 × 1 × 1 convolution, with all channels recombined to form complete cross‐modal feature maps. Finally, an Enhanced Position Attention (EPA) mechanism is designed to generate spatially adaptive weights by learning global positional dependencies, enhancing modal features with stronger discriminative power in key regions such as vertebral boundaries.

(2) Graph‐based structural representation learning

The graph‐based structural representation learning module explicitly introduces anatomical prior knowledge to maintain topological consistency among vertebral structures. We model the lumbar spine anatomy as a graph G = (V, E) with 10 nodes representing distinct anatomical structures: one background class (node 0), five lumbar vertebrae L1‐L5 (nodes 1–5), and four intervertebral discs L1/L2, L2/L3, L3/L4, and L4/L5 (nodes 6–9). This node assignment directly corresponds to the anatomical segmentation classes in our task. The edge connectivity follows the anatomical principle that each vertebra is directly adjacent to its neighboring intervertebral discs. For example, the L2 vertebra (node 2) connects to both L1/L2 disc (node 6, superior) and L2/L3 disc (node 7, inferior), reflecting the natural sandwich structure of the spine. Similarly, L1 vertebra (node 1) connects only to L1/L2 disc (node 6) as it is the superior‐most lumbar vertebra. This bipartite connectivity pattern ensures accurate representation of functional spinal unit organization. The adjacency matrix is generated from the adjacency list through nx.adjacency_matrix conversion and preprocessed using symmetric normalization. We applied symmetric normalization with self‐loop augmentation:

(6) $$\tilde{A\;}=D^{-\frac{1}{2}}\;(A+I)D^{-\frac{1}{2}},$$

where I adds self‐loops during preprocessing to enable self‐feature aggregation. Self‐loops are not in the original adjacency definition but introduced here as standard GCN practice. D is the degree matrix, satisfying $D_{ii}=\sum_{j}{\left(A+I\right)}_{ij}$. This normalization strategy ensures numerical stability and smooth information propagation during the graph convolution process.

Deep features obtained from collaborative learning are initially transformed into graph node representations via the Featuremaps_to_Graph module:

(7) $$G=W_{f_{2}g}\;\mathrm{GAP}(F_{\mathrm{co}-\mathrm{learn}}),$$

whereGAP(·) represents the global adaptive average pooling operation, and $W_{f_{2}g}\in R^{H\times C}$ is the feature‐to‐graph‐node projection matrix.

Subsequently, the node features are iteratively updated through a three‐layer graph convolutional network:

(8) $$G^{(l+1)}=\mathrm{ReLU}(\tilde{A}G^{\left(l\right)}W^{\left(l\right)}),$$

where $W^{\left(l\right)}\in R^{H\times H}$ is the learnable weight matrix of the l‐th layer, enabling each node to fuse contextual information from adjacent anatomical structures.

This hierarchical propagation mechanism allows each node to progressively incorporate contextual information from neighboring anatomical structures: Layer 1 aggregates local information from direct neighbors, Layer 2 enables cross‐node information exchange, and Layer 3 imposes global spinal topology constraints. The updated graph features are subsequently projected back to spatial feature maps via the graph‐to‐feature transformation module and combined with original features through residual connections, thereby preserving anatomical continuity in the segmentation results.

#### 2.2.3 Depth‐aware progressive upsampling (DAPU)

To effectively recover high‐resolution details, this paper proposes a depth‐aware progressive upsampling module (DAPU), which achieves depth‐adaptive perception and feature progressive reconstruction through a multi‐branch collaborative mechanism. Depth attention enhancement performs depth‐dimensional modeling on input features, extracting depth‐specific features through adaptive average pooling that preserves the depth dimension.

**FIGURE 3:**
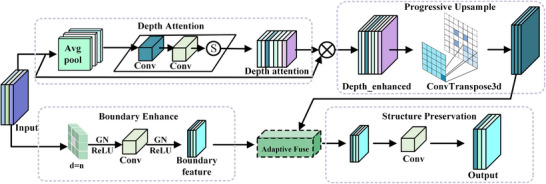
Depth‐aware progressive upsampling (DAPU) module with depth attention, boundary enhancement, progressive upsampling, and structure preservation components for 3D medical image reconstruction.

As depicted in Figure 3, the DAPU module contains four core components: depth attention, boundary enhancement, progressive upsampling, and structure preservation. Given an input feature map $X\in R^{C\times D\times H\times W}$, where C denotes the number of channels, D represents the depth dimension (number of slices along the axial direction), H represents the height dimension, and W represents the width dimension, the depth attention branch targets the characteristics of 3D data by learning slice importance weights through pooling operations that maintain the depth dimension. For input feature X, the depth perception process is:

(9) $$F_{\mathrm{depth}}=\frac{1}{HW}\sum_{h=1}^{H}\sum_{w\;=1}^{W}X_{:,:,:,h,w},$$

(10) $$A_{d}=\sigma (W_{2}\cdot \mathrm{ReLU}(W_{1}\cdot F_{\mathrm{depth}}+b_{1})+b_{2}),$$

where $F_{\mathrm{depth}}$ maintains depth dimension integrity, and $W_{1}$ and $W_{2}$ are channel compression and recovery weights, respectively. The depth‐enhanced feature is:

(11) $$X_{\mathrm{depth}\_\mathrm{enh}}=A_{d}⊙X,$$

where ⊙ denotes element‐wise multiplication, and $A_{d}$ is broadcast along the H and W dimensions to match the shape of X. This operation enables the network to selectively emphasize informative depth slices while suppressing less relevant ones, thereby enhancing the representation of anatomically significant structures across different axial positions.

In parallel, boundary feature extraction employs a multi‐scale dilated convolution strategy to capture anatomical structure boundary information, extracting multi‐scale boundary features through convolution kernels with different dilation rates. The progressive upsampling stage performs step‐by‐step reconstruction of depth‐enhanced features. Adaptive feature fusion combines the progressive upsampling results with interpolated boundary features, achieving effective integration of multi‐source information through concatenation operations and convolutional layers, and finally generates output through structure‐preserving convolution and skip connections.

#### 2.2.4 Loss function

We proposed a novel hybrid loss function specifically designed for three‐dimensional spinal magnetic resonance image segmentation. The Hybrid Frequency‐Domain Aware Tversky Loss (HFD‐Tversky) is optimally designed for the different anatomical characteristics of vertebrae and intervertebral discs, effectively addressing common challenges in spinal segmentation such as class imbalance and boundary identification difficulties. The HFD‐Tversky loss function can be expressed as:

(12) $$L_{\mathrm{HFD}-\mathrm{Tversky}}=\lambda \cdot L_{\mathrm{Tversky}}+(1-\lambda )\cdot L_{\mathrm{FD}-\mathrm{Tversky}},$$

where λ is the hybrid ratio coefficient, $L_{\mathrm{Tversky}}$ is the Tversky loss, and $L_{\mathrm{FD}-\mathrm{Tversky}}$ is the frequency‐domain aware Tversky loss.

The Tversky loss applies to all classes, with particular emphasis on the segmentation accuracy of intervertebral discs:

(13) $$\begin{array}{cc} L_{\mathrm{Tversky}} & =\frac{1}{\sum_{c}w_{c}}\sum_{c\;=0}^{C-1}w_{c}\\ & \;\cdot \left(1-\frac{\sum_{i}p_{i,c}\cdot g_{i,c}+\varepsilon }{\sum_{i}p_{i,c}\cdot g_{i,c}+\alpha_{c}\cdot \mathrm{FP}+\beta_{c}\cdot \mathrm{FN}+\varepsilon }\right), \end{array}$$

where $w_{c}$ is the weight of class c, $p_{i,c}$ and $g_{i,c}$ are the predicted probability and ground truth label of class c at position i, respectively, $\alpha_{c}$ and $\beta_{c}$ are the false positive (FP) and false negative (FN) weights of class c, the weight of $\beta_{c}$ for the intervertebral disc class is increased by 20% to enhance the penalty for missing intervertebral discs. ε is the smoothing term to prevent division by zero errors.

The frequency‐domain aware Tversky loss is primarily designed for vertebrae, combining spatial Tversky loss and frequency‐domain gradient loss:

(14) $$L_{\mathrm{FD}-\mathrm{Tversky}}{=L}_{\mathrm{Tversky}}^{\mathrm{vert}}+\omega \cdot L_{\mathrm{Freq}},$$

where $L_{\mathrm{Tversky}}^{\mathrm{vert}}$ is the Tversky loss specifically for vertebrae, ω is the frequency‐domain loss weight, and $L_{\mathrm{Freq}}$ is the frequency‐domain gradient loss, defined as:

(15) $$L_{\mathrm{Freq}}=\frac{1}{N}\sum_{i}∥\nabla p_{i}^{\mathrm{vert}}-\nabla g_{i}^{\mathrm{vert}}∥,$$

$\nabla p_{i}^{\mathrm{vert}}$ and $\nabla g_{i}^{\mathrm{vert}}$ represent the three‐dimensional gradient magnitudes of vertebral prediction and ground truth labels at position i, respectively, obtained by calculating the average of gradients in the depth, height, and width directions. Frequency domain gradient loss can effectively capture the structural boundary information of vertebrae, enhance the model's perception ability of vertebral morphology, and have significant advantages for recognizing the complex geometric shapes of vertebrae.

### 2.3 Implementation details

During the experimental process, the initial learning rate was set to 0.001. The network was trained using the HFD‐Tversky loss function and Adam optimizer. A learning rate decay strategy was applied, reducing the learning rate to 80% of its current value every 30 epochs. In the graph convolutional neural network segmentation module, a three‐layer graph convolutional neural network was employed for global information propagation, with the graph node feature dimension set to 128.

This study adopted the PyTorch framework for model training. Input data were processed in floating‐point tensor format, while target labels were converted to one‐hot encoding format, containing three categories: background, vertebral body, and intervertebral disc. During the training process, we implemented a deep supervision mechanism, with the decay coefficient (alpha) initial value of 0.4, decaying by 0.8 times every 30 epochs. To prevent overfitting, we introduced an early stopping mechanism by monitoring the average validation Dice score for vertebral body and intervertebral disc segmentation. All experiments were conducted on a workstation equipped with a Tesla V100‐PCIE 32GB GPU, a 24‐core Xeon Platinum 8268 CPU, and 64 GB RAM. The average inference time, measured on the test set, was 14 seconds per case.

### 2.4 Evaluation metrics

Dice Similarity Coefficient (DSC), Intersection over Union (IoU), 95th Percentile Hausdorff Distance (HD95), and Average Symmetric Surface Distance (ASSD) are used to quantitatively evaluate the segmentation performance of our proposed method.

(16) $$DSC=\frac{2\times |GT\cap P\mathrm{red}|+1}{|GT\left|+\right|P\mathrm{red}|+1},$$

(17) $$IoU=\frac{|GT\cap Pred|+1}{|GT\left|+\right|Pred\left|-\right|GT\cap Pred|+1},$$

(18) $$HD_{95}(Y,\;P)=max\{d_{\mathrm{YP}},\;d_{\mathrm{PY}}\},$$

(19) $$\begin{array}{cc} ASSD(A,B) & =\frac{1}{\left|S(A)+S(B)\right|}\left(\sum_{s_{A}\in S\left(A\right)}d\left(s_{A},S\left(B\right)\right)\right.\\ & \;\left.+\sum_{s_{B}\in S\left(B\right)}d\left(s_{B},S\left(A\right)\right)\right), \end{array}$$

where GT represents the ground truth annotation of the input image, and Pred represents the predicted segmentation result of the network. Y is the voxel set of the ground truth mask, P is the voxel set of the segmentation result boundary, $d_{YP}$ is the maximum 95% distance from segmentation result boundary voxels to ground truth mask voxels, $d_{PY}$ is the maximum 95% distance from ground truth mask voxels to segmentation result boundary voxels. A is the ground truth, B is the prediction, S(A) denotes the surface voxel set of the ground truth, and S(B) denotes the surface voxel set of the prediction. $s_{A}$ is an element in S(A), $s_{B}$ is an element in S(B).

## 3 RESULTS

### 3.1 Comparative experiments

#### 3.1.1 Evaluation on the normal test set

**FIGURE 4:**
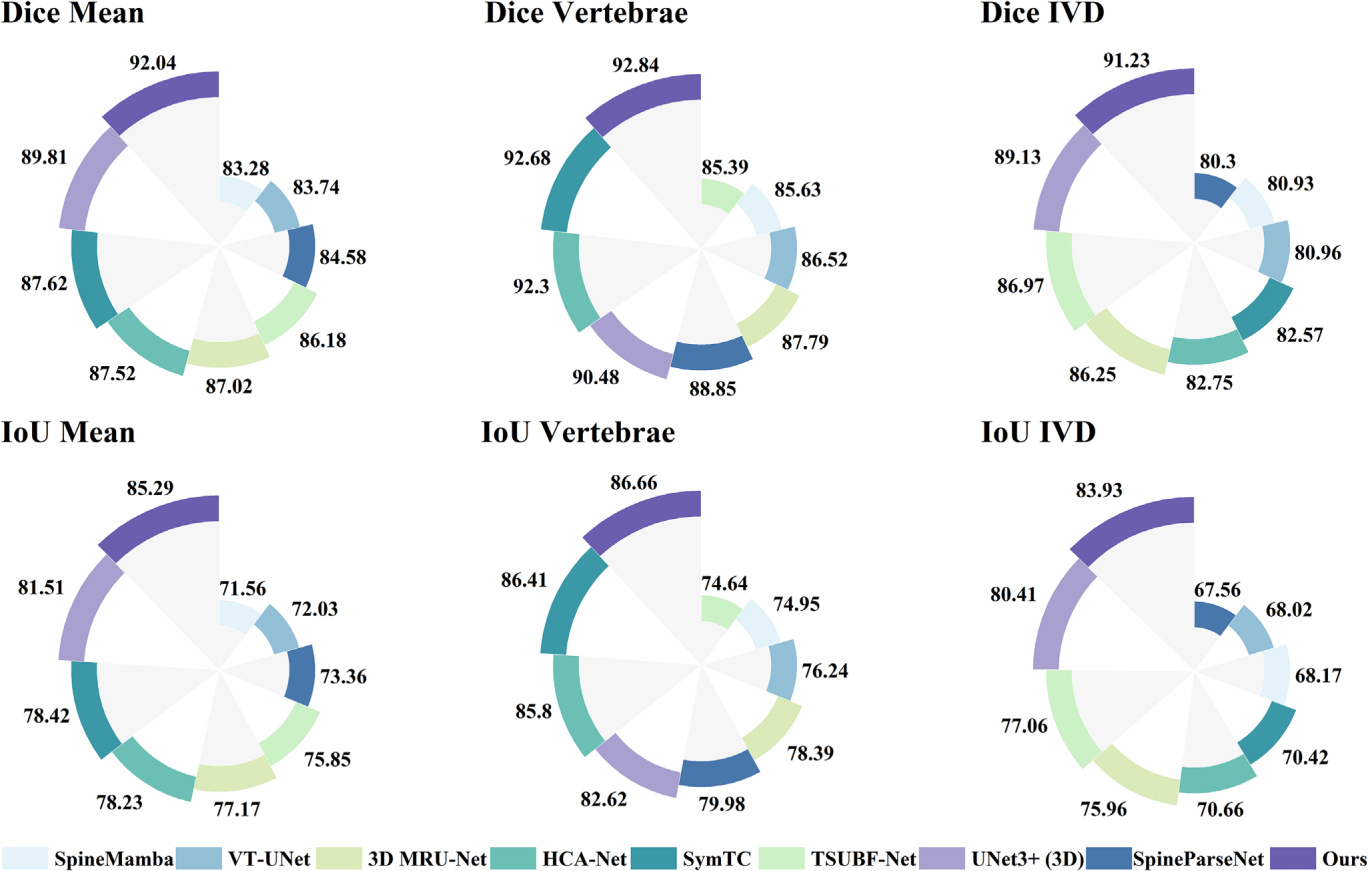
Comparison of segmentation performance metrics. Top row: Dice Similarity Coefficient (DSC) for Mean, Vertebrae, and Intervertebral Disc (IVD) segmentation. Bottom row: IoU metrics for Mean, Vertebrae, and Intervertebral Disc segmentation. Different colors represent various segmentation methods as indicated in the legend.

This study conducted comparative experiments with multiple advanced methods on lumbar spine MR image segmentation tasks. From the quantitative results in Table 1 and Figure 4, it can be observed that the proposed GF‐SSNet demonstrates excellent performance across all evaluation metrics, achieving 92.04 ± 0.06%, 92.84 ± 0.07%, and 91.23 ± 0.08% on the three key indicators of Dice Mean, Dice Vertebrae, and Dice IVD, respectively, significantly outperforming other comparative methods. In terms of IoU metrics, this method also maintains a leading position. Compared to the traditional U‐Net architecture, the dual‐branch encoder design of this method can better handle multimodal information fusion, avoiding the limitations of single feature representation. Compared to methods such as UNet3+(3D),^11^ 3D MRU‐Net,^28^ and SpineParseNet,^29^ the Mamba mechanism introduced in this study demonstrates higher computational efficiency and stronger long‐range modeling capability when processing 3D sequential data, which enables the model to achieve improvements in Dice coefficient. Compared to Transformer‐based methods such as VT‐UNet^30^ and TSUBF‐Net,^31^ the linear complexity characteristics of Mamba enable this method to significantly reduce computational overhead while maintaining high accuracy, surpassing VT‐UNet in IoU metrics. Particularly noteworthy is that compared to existing Mamba variants such as SpineMamba,^32^ this method achieves significant improvement in HD95 metrics through the combination of multi‐directional sequence modeling and graph convolutional networks, fully demonstrating the effectiveness of the architectural design. The advantages of this method over advanced methods such as SymTC^15^ and HCA‐Net^33^ are mainly reflected in the effective utilization of anatomical structure prior knowledge. By explicitly modeling the spatial relationships between vertebrae through graph convolutional networks, the segmentation results better conform to medical anatomical principles.

**TABLE 1:** Quantitative comparison of DSC and IoU metrics on the normal lumbar spine test set.

	DSC (%)			IoU (%)		
Models	Mean↑	Vertebrae↑	IVD↑	Mean↑	Vertebrae↑	IVD↑
UNet3+ (3D)	89.81 ± 0.61	90.48 ± 0.58	89.13 ± 0.72	81.51 ± 0.91	82.62 ± 1.02	80.41 ± 1.17
SpineParseNet	84.58 ± 2.63	88.85 ± 1.56	80.30 ± 6.44	73.36 ± 3.90	79.98 ± 2.53	67.56 ± 8.69
VT‐UNet	83.74 ± 0.64	86.52 ± 0.38	80.96 ± 0.90	72.03 ± 0.95	76.24 ± 0.59	68.02 ± 1.28
HCA‐Net	87.52 ± 0.56	92.30 ± 0.58	82.75 ± 0.54	78.23 ± 0.84	85.80 ± 0.94	70.66 ± 0.75
3D MRU‐Net	87.02 ± 0.20	87.79 ± 0.23	86.25 ± 0.24	77.17 ± 0.31	78.39 ± 0.34	75.96 ± 0.38
SymTC	87.62 ± 0.09	92.68 ± 0.09	82.57 ± 0.28	78.42 ± 0.14	86.38 ± 0.15	70.42 ± 0.44
SpineMamba	83.28 ± 0.23	85.63 ± 0.60	80.93 ± 1.04	71.56 ± 0.28	74.95 ± 0.91	68.17 ± 1.48
TSUBF‐Net	86.18 ± 0.01	85.39 ± 0.01	86.97 ± 0.01	75.85 ± 0.01	74.64 ± 0.01	77.06 ± 0.01
Ours (T1)	90.74 ± 0.14	91.86 ± 0.14	89.63 ± 0.22	83.14 ± 0.22	85.01 ± 0.22	81.28 ± 0.22
Ours (T2)	91.7 ± 0.09	92.52 ± 0.07	90.88 ± 0.11	84.76 ± 0.13	86.16 ± 0.15	83.37 ± 0.014
**Ours (T1+T2)**	**92.04 ± 0.06**	**92.84 ± 0.07**	**91.23 ± 0.08**	**85.29 ± 0.1**	**86.66 ± 0.11**	**83.93 ± 0.13**

*Note*: The best results are highlighted in bold.

Abbreviations: DSC, dice similarity coefficient; IoU, intersection over union; IVD, intervertebral disc.

**FIGURE 5:**
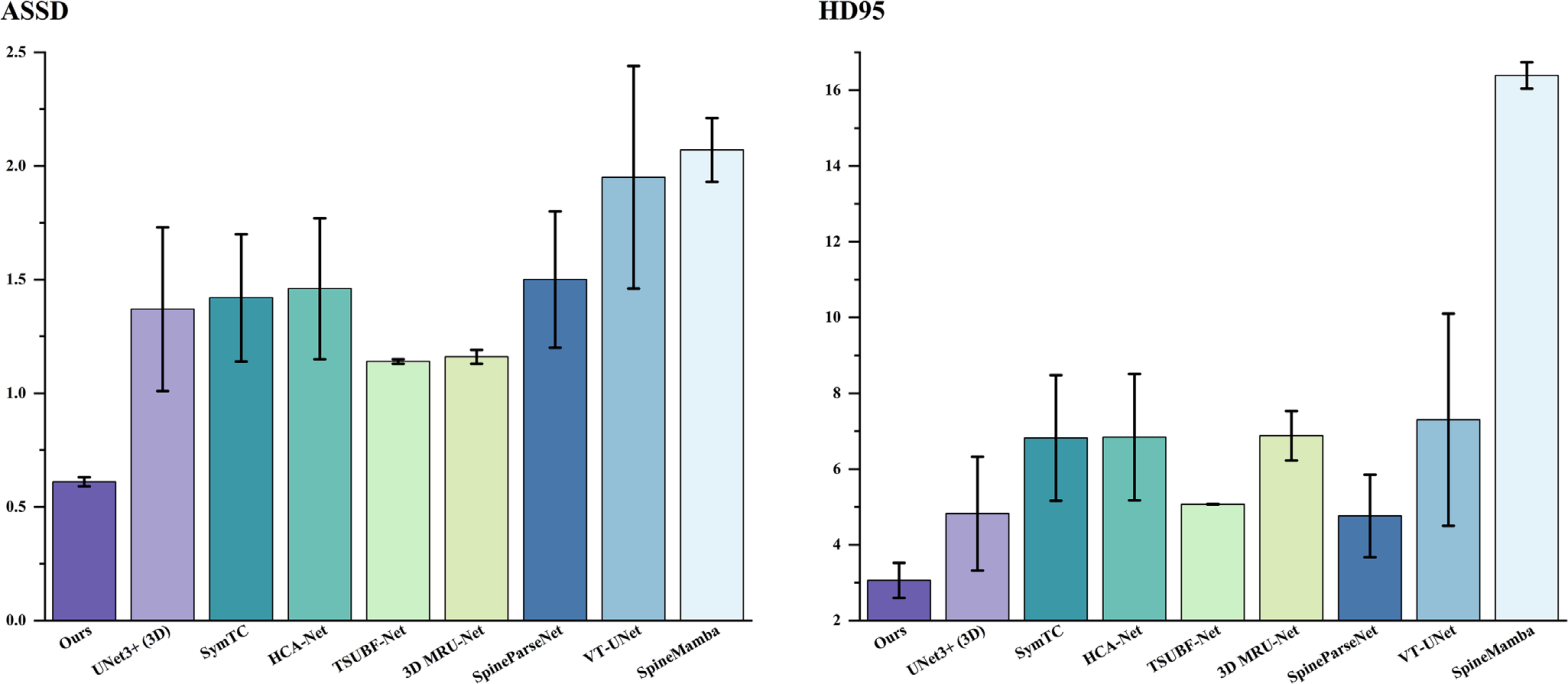
Quantitative comparison of different segmentation methods for lumbar spine structures, with error bars representing standard deviation. Left figure: Performance measured using the ASSD metric. Right figure: Performance measured using the HD95 metric. The x‐axis lists various segmentation methods.

Table 2 and Figure 5 further demonstrate the distance metric results. Compared to the best baseline methods, GF‐SSNet significantly reduced the HD95 by 1.70 mm (3.06 mm vs. 4.76 mm for SpineParseNet) and the ASSD by 0.53 mm (0.61 mm vs. 1.14 mm for TSUBF‐Net), demonstrating its advantages in boundary accuracy and overall segmentation quality. These lower HD95 and ASSD values indicate that the segmentation results produced by this method exhibit higher clinical usability and can provide clinicians with more accurate anatomical structure information to assist diagnostic decision‐making.

**TABLE 2:** Quantitative comparison of HD95 and ASSD metrics on the normal lumbar spine test set.

Models	HD95 (mm)↓	ASSD (mm)↓
UNet3+ (3D)	4.82 ± 1.50	1.37 ± 0.36
SpineParseNet	4.76 ± 1.09	1.50 ± 0.30
VT‐UNet	7.30 ± 2.80	1.95 ± 0.49
HCA‐Net	6.84 ± 1.67	1.46 ± 0.31
3D MRU‐Net	6.88 ± 0.65	1.16 ± 0.03
SymTC	6.82 ± 1.66	1.42 ± 0.28
SpineMamba	16.39 ± 0.35	2.07 ± 0.14
TSUBF‐Net	5.07 ± 0.01	1.14 ± 0.01
Ours (T1)	3.89 ± 0.06	0.71 ± 0.01
Ours (T2)	3.63 ± 0.09	0.66 ± 0.01
**Ours (T1+T2)**	**3.06 ± 0.46**	**0.612 ± 0.018**

*Note*: the best results are highlighted in bold.

Abbreviations: ASSD, average symmetric surface distance; HD95, 95th percentile Hausdorff distance.

**FIGURE 6:**
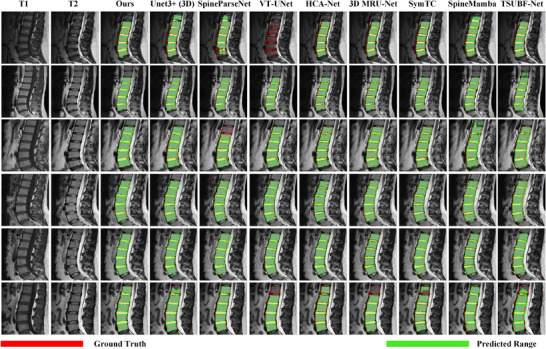
Visual comparison results of different methods on lumbar spine MRI segmentation tasks. The first and second columns show T1 and T2 weighted original MRI images, respectively, the third column shows the segmentation results of the GF‐SSNet method proposed in this paper, and the remaining columns show the segmentation results of various comparison methods. Each row displays the segmentation effects of different cases, where red regions represent Ground Truth and green regions represent Predicted Range. When red regions appear, it indicates that the method has under‐segmentation phenomena.

As shown in Figure 6, the visual comparison results demonstrate that GF‐SSNet achieves significant advantages in segmenting complex anatomical structures. Specifically, UNet3+ (3D) exhibits obvious over‐segmentation in row 1, where the green region substantially exceeds the true vertebral boundaries, while displaying under‐segmentation in row 6 with red areas remaining unidentified. SpineParseNet performs poorly, showing extensive red under‐segmentation in rows 1 and 3, resulting in severely incomplete vertebral structure identification. VT‐UNet demonstrates substantial segmentation failures in row 1, barely identifying any vertebral structures, and also presents notable under‐segmentation issues in row 6. Although HCA‐Net performs relatively stable in some cases, it still shows red under‐segmentation areas in row 6. While 3D MRU‐Net and SymTC show relatively stable overall performance, they still have locally imprecise issues in detail boundary processing. SpineMamba and TSUBF‐Net mainly exhibit over‐segmentation tendencies, with green areas exceeding true anatomical boundaries observable in multiple cases, incorrectly identifying non‐target areas as vertebral or intervertebral disc structures.

These visual improvements are primarily attributed to the synergistic integration of the dual‐branch encoding architecture, FDConv‐based frequency feature extraction, and Mamba‐based long‐range dependency modeling, further refined by GCN‐guided anatomical priors and the specialized HFD‐Tversky loss function. This comprehensive framework demonstrates greater potential for maintaining robust performance and boundary precision in increasingly complex clinical scenarios.

#### 3.1.2 Evaluation on the pathological test set

To assess the clinical applicability and robustness of the proposed method across various spinal pathologies, we conducted additional experiments on an independent pathological test set. This test set was constructed specifically to include cases with degenerative spinal conditions such as disc herniation, spinal stenosis, and degenerative disc disease, which are commonly encountered in clinical practice.

As shown in Table 3, we evaluated all comparison methods on this independent pathological test set. Compared to the normal test set (Table 1), all methods exhibited performance degradation on pathological cases, reflecting the increased complexity introduced by anatomical variations and structural abnormalities. Our method achieved an average Dice score of 87.60 ± 0.10%, representing a decrease of approximately 4.4 percentage points from the normal test set performance (92.04 ± 0.06%).

**TABLE 3:** Quantitative comparison of comprehensive metrics on the independent pathological test set.

	DSC (%)			IoU (%)				
Models	Mean↑	Vertebrae↑	IVD↑	Mean↑	Vertebrae↑	IVD↑	HD95 (mm)↓	ASSD (mm)↓
UNet3+ (3D)	38.07 ± 1.70	47.84 ± 1.77	28.30 ± 1.63	27.54 ± 1.35	34.28 ± 1.46	20.8 ± 1.04	48.84 ± 2.73	12.18 ± 2.30
SpineParseNet	73.08 ± 3.25	80.46 ± 1.24	65.71 ± 5.28	59.05 ± 3.11	67.69 ± 1.57	50.41 ± 4.64	18.83 ± 0.65	2.73 ± 0.34
VT‐UNet	73.04 ± 0.54	76.72 ± 0.23	69.36 ± 0.83	61.17 ± 0.75	66.56 ± 0.36	55.78 ± 1.15	9.50 ± 0.23	2.17 ± 0.04
3D MRU‐Net	81.67 ± 0.06	86.34 ± 0.47	77.01 ± 0.35	69.57 ± 0.13	76.14 ± 0.67	63.00 ± 0.42	8.17 ± 2.07	1.53 ± 0.17
SymTC	78.92 ± 0.18	85.70 ± 0.06	72.15 ± 0.30	66.08 ± 0.25	75.08 ± 0.10	57.08 ± 0.41	16.84 ± 0.32	2.27 ± 0.02
SpineMamba	75.10 ± 1.75	81.38 ± 0.26	68.81 ± 3.22	60.77 ± 2.04	68.67 ± 0.37	52.88 ± 3.71	20.40 ± 1.06	2.81 ± 0.22
TSUBF‐Net	82.03 ± 0.01	83.44 ± 0.12	80.53 ± 0.01	69.68 ± 0.02	71.78 ± 0.03	67.57 ± 0.06	5.10 ± 0.13	1.34 ± 0.01
**Ours**	**87.60 ± 0.10**	**90.87 ± 0.10**	**84.33 ± 0.11**	**78.13 ± 0.15**	**83.29 ± 0.15**	**72.97 ± 0.16**	**3.55 ± 0.10**	**0.84 ± 0.01**

*Note*: The best results are highlighted in bold.

Notably, the performance decline was more pronounced for intervertebral disc (IVD) segmentation compared to vertebrae segmentation. Specifically, IVD Dice decreased from 91.23 ± 0.08% to 84.33 ± 0.11% (a decline of approximately 6.9 percentage points), while Vertebrae Dice decreased from 92.84 ± 0.07% to 90.87 ± 0.10% (a decline of approximately 2.0 percentage points). This differential degradation suggests that IVD segmentation is more susceptible to pathological variations, likely because degenerative changes such as disc herniation, height loss, and signal intensity alterations primarily affect the disc structure, leading to greater ambiguity in boundary identification and increased segmentation difficulty.

While the performance gap between normal and pathological cases, particularly for IVD segmentation, indicates room for improvement in handling complex spinal pathologies, the results demonstrate that our approach maintains competitive performance relative to existing methods on clinically challenging cases. The observed performance pattern suggests that the proposed architectural design provides some robustness to anatomical variations, though the more significant degradation in IVD segmentation highlights the need for targeted strategies to better capture pathological disc features in future work. These findings provide preliminary evidence for the potential clinical utility of the method, while identifying specific areas for enhancement, particularly in addressing degenerative disc pathologies.

#### 3.1.3 Error analysis and failure cases

To comprehensively evaluate the method's limitations, we analyzed the worst‐performing cases from both test sets. Representative challenging cases were identified by selecting slices with the lowest Dice scores. For each case, three complementary visualizations were generated: ground truth versus prediction overlays, voxel‐wise error maps, and surface distance error maps.

**FIGURE 7:**
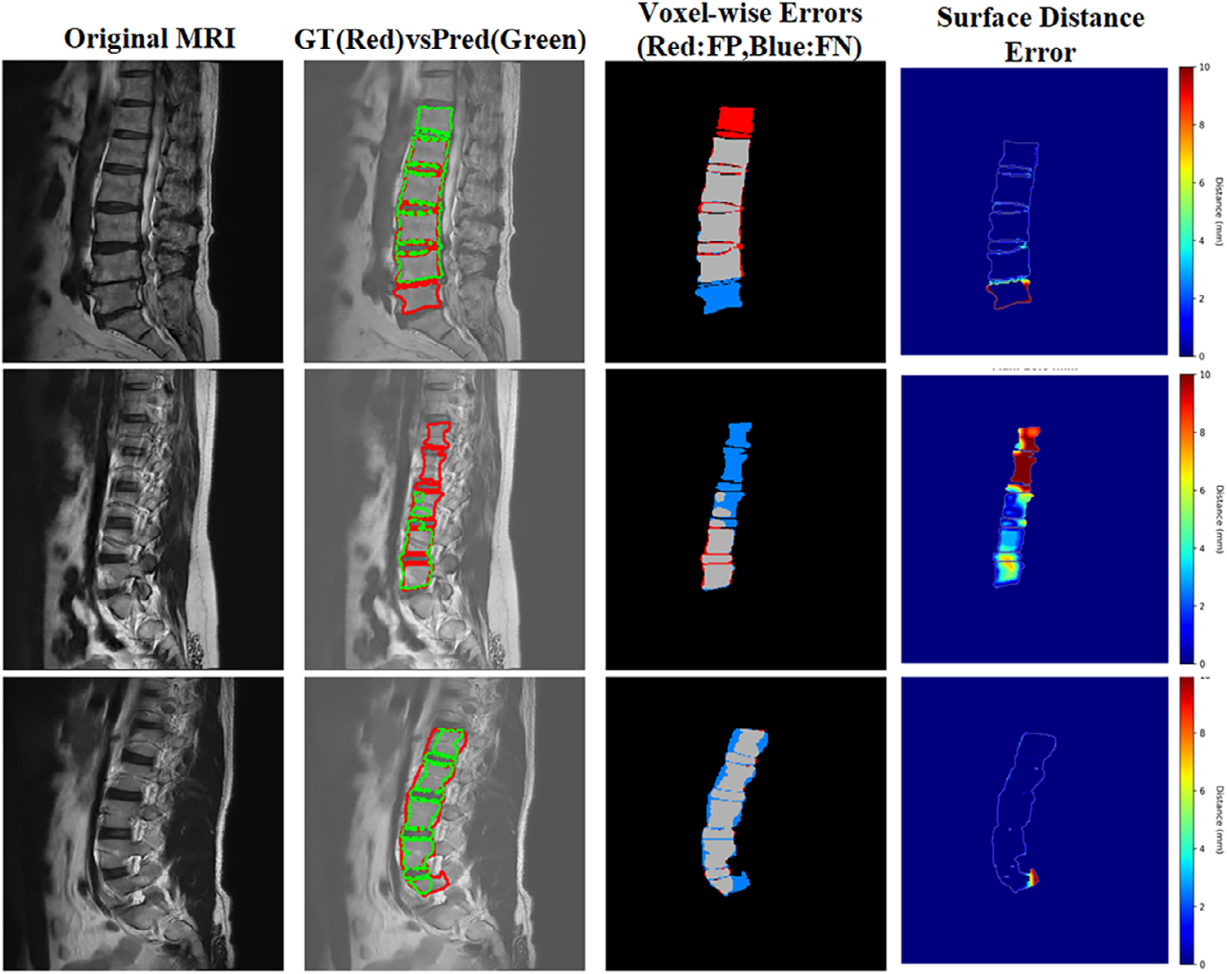
Error analysis of worst‐performing cases. (Column 1) Original MRI; (Column 2) Ground truth (red) vs. prediction (green); (Column 3) Voxel‐wise errors (red: false positive, blue: false negative); (Column 4) Surface distance errors (mm). The top row shows failures in severe disc degeneration; the bottom rows show errors in edge slices with fragmentary structures. Errors remain localized despite being the worst cases.

Through systematic examination of these challenging cases, we identify two primary sources of segmentation difficulty. The most challenging scenarios occur in cases with severe disc degeneration where pathological changes cause substantial anatomical distortions that deviate from normal training distributions. As shown in Figure 7 (top row), disc height loss and endplate irregularities create anatomical configurations underrepresented in training data, leading to boundary delineation errors at the intervertebral disc interface. The second challenging scenario mode occurs in edge slices with fragmentary vertebral structures. When only partial anatomical information is visible, the model exhibits reduced confidence in boundary localization (Figure 7, middle and bottom rows). As demonstrated in both cases, the progressive reduction in visible vertebral anatomy—from partial vertebral body fragments to minimal structural features—correlates with increasing segmentation uncertainty and boundary localization errors.

Importantly, surface distance error maps reveal that even in the most challenging cases, errors are highly localized. The vast majority of structures show minimal error, with errors concentrated in small regions of severe pathological change or structural ambiguity rather than representing widespread segmentation failure. These error patterns reflect inherent challenges in medical image segmentation driven by extreme pathological variations and limited anatomical context. Understanding these patterns provides valuable insights for future improvements through targeted data collection strategies. Moreover, this analysis informs clinical deployment strategies, such as implementing automated quality control mechanisms to flag cases requiring manual review.

### 3.2 Ablation experiments

#### 3.2.1 Model components

To validate the effectiveness of each key module in the proposed method, we conducted a detailed ablation experimental analysis. The results demonstrate that each core module contributed to the final performance, with strong synergistic effects observed among the modules.

As demonstrated in Table 4, the progressive incorporation of each module resulted in consistent performance improvements. The initial integration of the FDConv module improved the average Dice score to 90.36 ± 0.20%, demonstrating that the adaptive convolution kernel weighting mechanism effectively strengthens feature extraction capabilities. Subsequently, the incorporation of DAPU further elevated the average Dice score to 91.50 ± 0.10%, confirming the critical importance of the progressive upsampling strategy in preserving fine‐grained details.

**TABLE 4:** Quantitative comparison of individual architectural modules on the segmentation performance.

				Sag‐mamba	DSC (%)			IoU (%)				
FDConv	DAPU	TD‐Mamba	GCN		Mean↑	Vertebrae↑	IVD↑	Mean↑	Vertebrae↑	IVD↑	HD95 (mm)↓	ASSD (mm)↓
–	–	–	–	–	89.81 ± 0.61	90.48 ± 0.58	89.13 ± 0.72	81.51 ± 0.91	82.62 ± 1.02	80.41 ± 1.17	3.77 ± 0.20	0.71 ± 0.02
√	–	–	–	–	90.36 ± 0.20	91.44 ± 0.01	89.27 ± 0.40	82.51 ± 0.32	84.30 ± 0.01	80.72 ± 0.64	4.12 ± 0.36	0.78 ± 0.03
√	√	–	–	–	91.50 ± 0.10	92.26 ± 0.05	90.75 ± 0.16	84.39 ± 0.16	85.66 ± 0.09	83.13 ± 0.26	4.52 ± 0.27	0.72 ± 0.02
√	√	√	–	–	91.72 ± 0.12	92.67 ± 0.12	90.76 ± 0.12	84.77 ± 0.19	86.38 ± 0.19	83.16 ± 0.19	3.27 ± 0.20	0.64 ± 0.02
√	√	–	√	√	91.33 ± 0.38	92.11 ± 0.29	90.55 ± 0.49	84.13 ± 0.59	85.46 ± 0.47	82.82 ± 0.77	4.04 ± 0.58	0.70 ± 0.05
**√**	**√**	**√**	**√**	–	**92.04 ± 0.06**	**92.84 ± 0.07**	**91.23 ± 0.08**	**85.29 ± 0.1**	**86.66 ± 0.11**	**83.93 ± 0.13**	**3.06 ± 0.46**	**0.612 ± 0.018**

*Note*: the best results are highlighted in bold.

The incorporation of the Mamba module yielded substantial performance gains, with particularly notable improvements in distance‐based metrics. Following the integration of TD‐Mamba, HD95 improved from 4.52 ± 0.27 mm to 3.27 ± 0.20 mm, while ASSD decreased from 0.72 ± 0.02 mm to 0.64 ± 0.02 mm, highlighting the effectiveness of sequence modeling in enhancing segmentation boundary precision. The final incorporation of GCN brought the complete model to peak performance, achieving an average Dice score of 92.04 ± 0.06%, with HD95 further reduced to 3.06 ± 0.46 mm and ASSD to 0.612 ± 0.018 mm. This demonstrates that by explicitly modeling spatial relationships among anatomical structures, the GCN module effectively enhances the anatomical consistency of segmentation outcomes. To further validate the necessity of the three‐directional design, we compared the performance of sagittal‐only Mamba (Sag‐mamba) with the full three‐directional Mamba (TD‐mamba). As detailed in Table 4, while the sagittal direction captures substantial spinal spatial information, the TD‐mamba configuration achieved superior performance with an average Dice score of 92.04 ± 0.06% compared to 91.33 ± 0.38% for Sag‐mamba, demonstrating that the integration of depth, height, and width directional information provides complementary spatial context that enhances segmentation accuracy. This confirms that multi‐directional sequence modeling is essential for comprehensively capturing the complex 3D spatial relationships in spinal structures.

**FIGURE 8:**
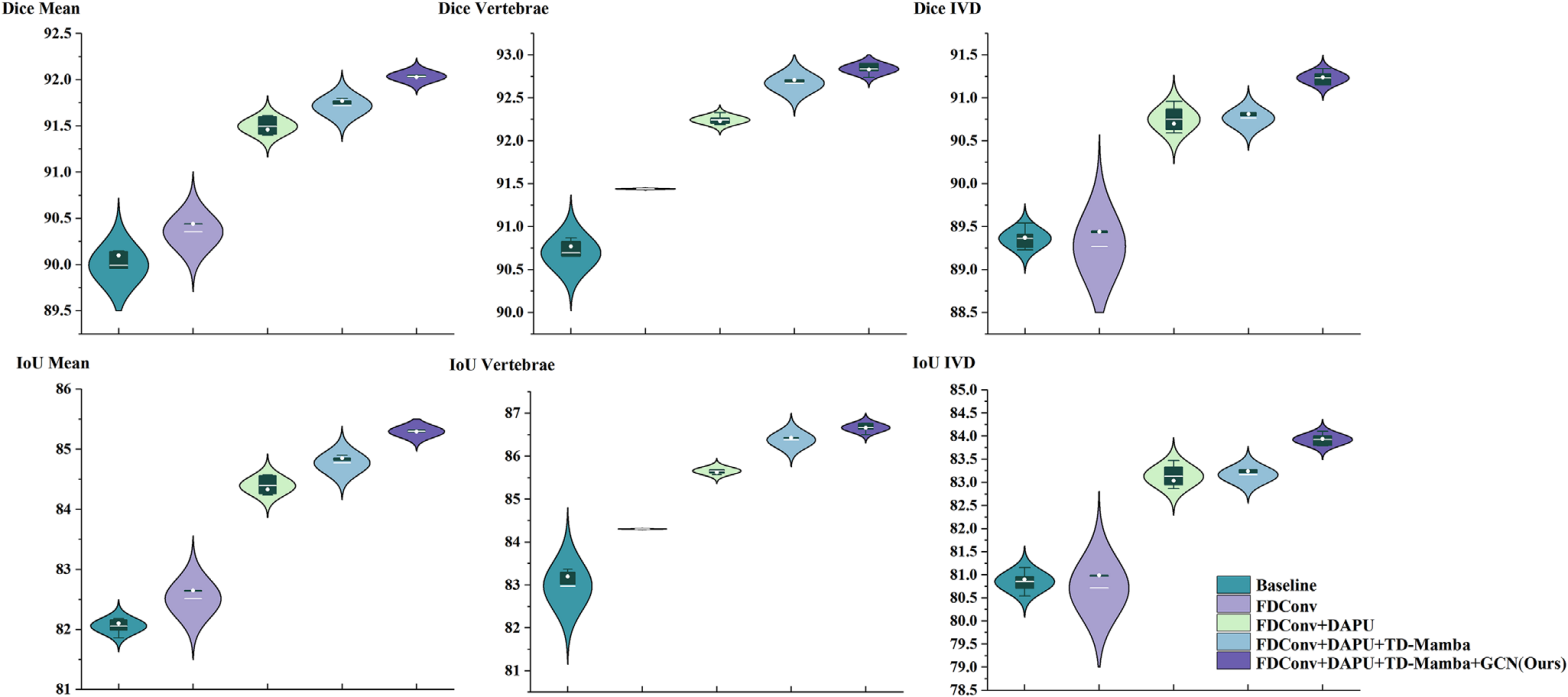
Violin box plot visualization of ablation experiment results for each module of GF‐SSNet, where the upper row shows DSC metrics and the lower row shows IoU metrics. The box plot portion shows the mean and interquartile range of the data, while the external contours reflect data probability density distributions, with different colors representing the performance of different module combinations.

From the distribution characteristics shown in the violin plot of Figure 8, the complete model achieves superior mean performance across all metrics, indicating that the synergistic integration of modules enhances both average performance and model robustness. As modules are progressively added from baseline to the complete architecture, we observe consistent improvements in both Dice and IoU metrics across different anatomical structures. Notably, the intervertebral disc segmentation demonstrates substantial improvement, underscoring the efficacy of the proposed architectural components in addressing complex anatomical structures. These ablation results confirm the validity of our proposed technical framework, with each module proving essential to overall performance gains.

**FIGURE 9:**
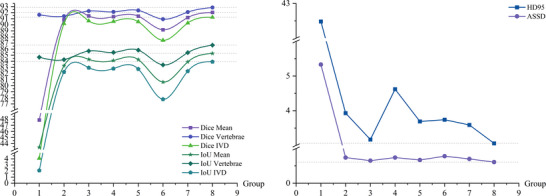
Impact of loss function parameter combinations on segmentation performance metrics. Left: DSC and IoU metrics; Right: HD95 and ASSD error metrics.

#### 3.2.2 Loss function

As presented in Tables 5 and 6, we conducted comprehensive comparisons of loss functions and parameter optimization analyses to further enhance model performance on lumbar spine segmentation. Table 5 compares the comparative performance of different loss functions, demonstrating that HFD‐Tversky loss achieves optimal performance across all evaluation metrics. Specifically, HFD‐Tversky attained a mean Dice score of 92.04 ± 0.06%, a mean IoU of 85.29 ± 0.1, HD95 of 3.06 ± 0.46 mm, and ASSD of 0.612 ± 0.018 mm. These results substantially outperform baseline methods.

**TABLE 5:** Quantitative comparison of different loss functions for lumbar spine MRI segmentation.

	DSC (%)			IoU (%)				
Loss	Mean↑	Vertebrae↑	IVD↑	Mean↑	Vertebrae↑	IVD↑	HD95 (mm)↓	ASSD (mm)↓
Dice	88.85 ± 0.07	90.71 ± 0.05	86.98 ± 0.11	80.03 ± 0.12	83.06 ± 0.09	77 ± 0.17	3.52 ± 0.04	0.801 ± 0.006
Cross Entropy	88.13 ± 0.18	90.01 ± 0.23	86.26 ± 0.58	78.91 ± 0.28	81.92 ± 0.37	75.91 ± 0.91	4.26 ± 0.28	0.868 ± 0.008
Focal	88.26 ± 0.08	89.80 ± 0.04	86.72 ± 0.14	79.11 ± 0.13	81.59 ± 0.07	76.64 ± 0.23	4.46 ± 0.02	0.878 ± 0.008
Tversky	90.82 ± 0.16	91.41 ± 0.07	90.20 ± 0.27	83.26 ± 0.27	84.28 ± 0.07	82.24 ± 0.46	3.93 ± 0.15	0.741 ± 0.013
FD‐Tversky	47.87 ± 0.09	91.64 ± 0.08	4.09 ± 0.12	43.38 ± 0.09	84.67 ± 0.13	2.09 ± 0.06	42.48 ± 1.04	5.333 ± 0.11
**HFD‐Tversky (Ours)**	**92.04 ± 0.06**	**92.84 ± 0.07**	**91.23 ± 0.08**	**85.29 ± 0.1**	**86.66 ± 0.11**	**83.93 ± 0.13**	**3.06 ± 0.46**	**0.612 ± 0.018**

*Note*: the best results are highlighted in bold.

Abbreviations: FD‐Tversky, frequency‐domain aware Tversky; HFD‐Tversky, hybrid frequency‐domain aware Tversky.

**TABLE 6:** Quantitative comparison of different parameter combinations for the HFD‐Tversky Loss.

Loss function parameters								DSC (%)			IoU(%)				
					n_weights										
Group	α	β	ω	λ	BG	Vertebrae	IVD	Mean↑	Vertebrae↑	IVD↑	Mean↑	Vertebrae↑	IVD↑	HD95 (mm)↓	ASSD (mm)↓
1	0.25	0.75	1	0	0.2	1.2	1.6	47.87 ± 0.09	91.64 ± 0.08	4.09 ± 0.12	43.38 ± 0.09	84.67 ± 0.13	2.09 ± 0.06	42.48 ± 1.04	5.333 ± 0.11
2	0.25	0.75	0	1	0.2	1.2	1.6	90.82 ± 0.16	91.41 ± 0.07	90.2 ± 0.27	83.26 ± 0.27	84.28 ± 0.07	82.24 ± 0.46	3.93 ± 0.15	0.741 ± 0.013
3	0.2	0.8	0.07	0.55	0.2	1.2	1.6	91.45 ± 0.02	92.25 ± 0.02	90.64 ± 0.02	84.32 ± 0.03	85.69 ± 0.04	82.95 ± 0.03	3.17 ± 0.04	0.65 ± 0.003
4	0.25	0.75	0.1	0.5	0.2	1.2	1.6	91.33 ± 0.11	92.12 ± 0.12	90.55 ± 0.10	84.13 ± 0.18	85.46 ± 0.20	82.80 ± 0.16	4.62 ± 0.53	0.744 ± 0.033
5	0.25	0.75	0.07	0.55	0.2	1.4	1.4	91.42 ± 0.04	92.36 ± 0.01	90.5 ± 0.06	84.28 ± 0.07	85.84 ± 0.07	82.73 ± 0.10	3.69 ± 0.11	0.67 ± 0.01
6	0.5	0.5	0.07	0.55	0.2	1.2	1.6	89.18 ± 0.04	90.92 ± 0.08	87.45 ± 0.07	80.59 ± 0.07	83.41 ± 0.13	77.77 ± 0.12	3.74 ± 0.28	0.784 ± 0.011
7	0.2	0.8	0.08	0.5	0.2	1.3	1.7	91.19 ± 0.07	92.09 ± 0.06	90.29 ± 0.11	83.90 ± 0.13	85.43 ± 0.10	82.38 ± 0.18	3.59 ± 0.28	0.700 ± 0.013
**8 (Ours)**	**0.25**	**0.75**	**0.07**	**0.55**	**0.2**	**1.2**	**1.6**	**92.04 ± 0.06**	**92.84 ± 0.07**	**91.23 ± 0.08**	**85.29 ± 0.1**	**86.66 ± 0.11**	**83.93 ± 0.13**	**3.06 ± 0.46**	**0.612 ± 0.018**

*Note*: The best results are highlighted in bold.

Abbreviation: BG, Background.

Traditional loss functions exhibit inherent limitations in medical image segmentation. Standard Dice loss shows limited efficacy for small targets like intervertebral discs. Cross‐entropy loss performs poorly due to class imbalance, achieving only 86.26 ± 0.58% for intervertebral disc segmentation. While Focal loss partially addresses class imbalance, the overall improvement remains modest. Conversely, Tversky loss achieves superior performance on imbalanced data through adjustable false positive and false negative weighting, elevating the average Dice score to 90.82 ± 0.16% and intervertebral disc segmentation to 90.20 ± 0.27%. Remarkably, pure frequency domain loss (FD‐Tversky with λ = 0) nearly fails on intervertebral disc segmentation, indicating that pure frequency domain constraints are overly restrictive. These findings underscore the necessity of hybrid approaches that balance frequency domain constraints with spatial domain objectives.

The parameter optimization results presented in Table 6 demonstrate that different parameter combinations significantly affect model performance. The optimal parameter configuration (Group 8) achieves superior performance through effective balancing of false positive and false negative penalties, along with appropriate frequency domain weights and mixing ratios. Furthermore, the trend analysis depicted in Figure 9 corroborates the importance of parameter selection, revealing that both excessively low and high mixing ratios result in performance degradation. In contrast, the λ = 0.55 configuration preserves the benefits of frequency domain constraints while mitigating over‐regularization issues.

The effectiveness of the HFD‐Tversky loss function manifests in three key aspects: First, assigning higher false negative weights for intervertebral discs effectively reduces missed detections of small targets; Second, integrating frequency domain constraints enhances vertebral boundary definition, reducing the HD95 metric by 0.87 mm compared to Tversky loss (3.06 mm vs. 3.93 mm); Finally, the adaptive class weighting mechanism optimizes learning balance across heterogeneous anatomical structures.

## 4 DISCUSSION

The proposed GF‐SSNet achieved significant performance improvements in lumbar spine MR image segmentation. Through detailed ablation experiments and comparative analysis, we gained deep insights into how each component contributes to overall performance. The ablation experiment results clearly demonstrated the progressive contributions of each technical module, with the baseline model achieving an average Dice score of 89.81 ± 0.61%, while the complete model reached 92.04 ± 0.06%. This 2.23 percentage point improvement resulted from the integration of several key technological innovations.

The introduction of the FDConv module yielded a 0.55 percentage point performance improvement, which stemmed from its adaptive weight selection mechanism that dynamically adjusts convolutional kernel parameters based on the frequency domain characteristics of different anatomical structures. Through multi‐weight parallel convolution and attention‐weighted fusion, it effectively enhanced the model's ability to distinguish between different tissue types. The addition of the DAPU module further improved performance by addressing information loss in traditional upsampling methods in 3D medical images. Through integrating a depth attention branch, a boundary enhancement branch, and a progressive upsampling strategy, particularly demonstrating excellent performance in the reconstruction of small targets such as intervertebral discs.

Although TD‐Mamba contributed modestly to the average Dice score, it showed outstanding performance in distance metrics, with HD95 decreasing from 4.52 ± 0.27 mm to 3.27 ± 0.20 mm, and ASSD decreasing from 0.72 ± 0.02 mm to 0.64 ± 0.02 mm. This reflects the core value of the Mamba mechanism: through 3D sequence modeling, it effectively captures the spatial continuity of spinal structures, producing more continuous and anatomically consistent segmentation results. The final incorporation of the GCN module enables the complete model to achieve optimal performance. By mapping feature maps to graph structures and utilizing the adjacency relationships of spinal anatomy for graph convolution processing, the model can enforce anatomical constraints, ensuring that segmentation results conform to medical knowledge.

The HFD‐Tversky loss function is designed to account for the different characteristics of vertebral bodies and intervertebral discs. For vertebral bodies, it applies Tversky loss with frequency domain constraints to enhance boundary clarity, while for intervertebral discs, it uses pure Tversky loss with increased false negative weights to improve detection sensitivity. The failure of pure frequency domain loss FD‐Tversky validates the harm of excessive constraints, while the hybrid strategy achieves optimal performance with λ = 0.55, indicating the necessity of balancing constraint strength and segmentation accuracy in medical image segmentation. The HFD‐Tversky loss shows significant improvements over traditional losses across all metrics, achieving the best performance in both segmentation accuracy (DSC, IoU) and boundary precision (HD95, ASSD), which fully demonstrates the effectiveness of targeted loss function design.

Despite the promising results achieved by GF‐SSNet, this study has several limitations. First, the model shows limited generalization to severe pathological cases, as the training data predominantly consists of healthy and mild degenerative spine images. As demonstrated in the failure case analysis, extreme anatomical distortions from advanced disc degeneration can lead to segmentation errors due to deviation from the training distribution. Second, performance degradation occurs at edge slices where insufficient anatomical context is available. Third, the integration of multiple advanced components increases computational complexity, potentially limiting real‐time clinical deployment. Finally, the dual‐modality requirement may restrict applicability in scenarios where only single‐sequence MRI is available. Future research will focus on collecting diverse pathological data to improve generalization, exploring lightweight architectures to reduce computational costs, and developing single‐modality variants to broaden clinical applicability.

## 5 CONCLUSION

The Graph‐guided Frequency‐enhanced State Space Network (GF‐SSNet) proposed in this study provides an innovative solution for lumbar spine MRI segmentation. This network effectively addresses key challenges in 3D medical image segmentation by integrating frequency domain enhancement, multi‐dimensional state space modeling, and anatomical structure prior knowledge.

Through systematic experimental evaluation, GF‐SSNet significantly outperforms existing state‐of‐the‐art across all metrics, validating the effectiveness of the proposed technical approach. This superior performance stems from several key innovations: the synergistic design of FDConv and TD‐Mamba enables precise modeling of complex lumbar spine structures, GCN ensures anatomical rationality of segmentation results, and the DAPU module effectively addresses the deficiencies of traditional methods in detail reconstruction. The targeted HFD‐Tversky loss further enhances the accuracy across diverse anatomical structures.

The primary contribution of this work is a comprehensive 3D medical image segmentation framework, providing valuable technical reference for the segmentation of complex anatomical structures. Future work will focus on extending the application scope to full spine segmentation, as well as deepening integration with clinical decision support systems, providing technical support for achieving more intelligent and personalized medical services.

## AUTHOR CONTRIBUTIONS

Linghui Hong, the principal investigator, managed data, developed methodologies, conducted analysis, validated methods, and wrote the first draft. Zhengchao Zhou was responsible for data collection and participated in the design of the model architecture. Wanbo Xu provided resources. Both Pingping Wang and Benzheng Wei provided funding for this research. All authors reviewed and approved the final manuscript.

## FUNDING

This work was supported by the National Natural Science Foundation of China [No. 62372280, 62402297], the Natural Science Foundation of Shandong Province [No. ZR2022QG051, ZR2023QF094, ZR2024MF139], the Special fund of Qilu Health and Health Leading Talents Training Project, and the Young Talent of Lifting engineering for Science and Technology in Shandong, China.

## CONFLICT OF INTEREST STATEMENT

The authors have no relevant conflicts of interest to disclose.

## ETHICS STATEMENT

The study was conducted in accordance with the Declaration of Helsinki and approved by the Ethics Committee of Qilu Hospital of Shandong University Dezhou Hospital. This study and all research are approved and conducted following relevant guidelines.

## Data Availability

Authors will share data upon request to the corresponding author.

## References

[1] Hoy D , March L , Brooks P , et al. The global burden of low back pain: estimates from the Global Burden of Disease 2010 study. Ann Rheumat Dis. 2014;73(6):968‐974. doi:10.1136/annrheumdis‐2013‐204428 24665116 10.1136/annrheumdis-2013-204428

[2] Vos T , Flaxman AD , Naghavi M , et al. Years lived with disability (YLDs) for 1160 sequelae of 289 diseases and injuries 1990–2010: a systematic analysis for the Global Burden of Disease Study 2010. The Lancet. 2012;380(9859):2163‐2196. doi:10.1016/S0140‐6736(12)61729‐2 10.1016/S0140-6736(12)61729-2 PMC6350784 23245607

[3] Andersson GB . Epidemiological features of chronic low‐back pain. The Lancet. 1999;354(9178):581‐585. doi:10.1016/S0140‐6736(99)01312‐4 10.1016/S0140-6736(99)01312-4 10470716

[4] Murray CJ , Vos T , Lozano R , et al. Disability‐adjusted life years (DALYs) for 291 diseases and injuries in 21 regions, 1990–2010. The Lancet. 2012;380(9859):2197‐2223. doi:10.1016/S0140‐6736(12)61689‐4 10.1016/S0140-6736(12)61689-4 23245608

[5] Pfirrmann CWA , Metzdorf A , Zanetti M , et al. Magnetic resonance classification of lumbar intervertebral disc degeneration. Spine. 2001;26(17):1873‐1878. doi:10.1097/00007632‐200109010‐00011 11568697 10.1097/00007632-200109010-00011

[6] Carrino JA , Lurie JD , Tosteson ANA , et al. Lumbar spine: reliability of MR imaging findings. Radiology. 2009;250(1):161‐170. doi:10.1148/radiol.2493071999 18955509 10.1148/radiol.2493071999 PMC2657480

[7] Sekuboyina A , Husseini ME , Bayat A , et al. VerSe: a vertebrae labelling and segmentation benchmark for multi‐detector CT images. Medical Image Analysis. 2021;73:102166. doi:10.1016/j.media.2021.102166 34340104 10.1016/j.media.2021.102166

[8] Ronneberger O , Fischer P , Brox T , U‐Net: convolutional networks for biomedical image segmentation. arXiv. Preprint posted online May 18, 2015. doi:10.48550/arXiv.1505.04597

[9] Isensee F , Jaeger PF , Kohl SAA , Petersen J , Maier‐Hein KH . nnU‐Net: a self‐configuring method for deep learning‐based biomedical image segmentation. Nat Methods. 2021;18(2):203‐211. doi:10.1038/s41592‐020‐01008‐z 33288961 10.1038/s41592-020-01008-z

[10] Zhou Z , Rahman Siddiquee MM , Tajbakhsh N , Liang J . UNet++: a nested U‐Net architecture for medical image segmentation. In: Stoyanov D , Taylor Z , Carneiro G , eds. Deep Learning in Medical Image Analysis and Multimodal Learning for Clinical Decision Support. Vol 11045. Lecture Notes in Computer Science. Springer International Publishing; 2018:3‐11. doi:10.1007/978‐3‐030‐00889‐5_1 10.1007/978-3-030-00889-5_1 PMC7329239 32613207

[11] Huang H , Lin L , Tong R , et al. UNet 3+: a Full‐Scale Connected UNet for Medical Image Segmentation. In: ICASSP 2020 ‐ 2020 IEEE International Conference on Acoustics, Speech and Signal Processing (ICASSP). IEEE; 2020:1055‐1059. doi:10.1109/ICASSP40776.2020.9053405 10.1109/ICASSP40776.2020.9053555 PMC7543994 33041676

[12] Chen J , Lu Y , Yu Q , et al. TransUNet: transformers make strong encoders for medical image segmentation. arXiv. Preprint posted online February 8, 2021. doi:10.48550/arXiv.2102.04306

[13] Hatamizadeh A , Tang Y , Nath V , et al. UNETR: transformers for 3D medical image segmentation. In: 2022 IEEE/CVF Winter Conference on Applications of Computer Vision (WACV). IEEE; 2022:1748‐1758. doi:10.1109/WACV51458.2022.00181

[14] Hatamizadeh A , Nath V , Tang Y , Yang D , Roth H , Xu D , SwinUNETR: swin transformers for semantic segmentation of brain tumors in MRI images. arXiv. Preprint posted online January 4, 2022. doi:10.48550/arXiv.2201.01266

[15] Chen J , Qian L , Ma L , Urakov T , Gu W , Liang L . SymTC: a symbiotic Transformer‐CNN net for instance segmentation of lumbar spine MRI. Computers in Biology and Medicine. 2024;179:108795. doi:10.1016/j.compbiomed.2024.108795 38955128 10.1016/j.compbiomed.2024.108795

[16] Gu A , Goel K , Ré C , Efficiently Modeling Long Sequences with Structured State Spaces. arXiv. Preprint posted online August 5, 2022. doi:10.48550/arXiv.2111.00396

[17] Gu A , Dao T , Mamba: linear‐Time Sequence Modeling with Selective State Spaces. arXiv. Preprint posted online May 31, 2024. doi:10.48550/arXiv.2312.00752

[18] Ma J , Li F , Wang B , U‐Mamba: enhancing Long‐range Dependency for Biomedical Image Segmentation. arXiv. Preprint posted online January 9, 2024. doi:10.48550/arXiv.2401.04722

[19] Xing Z , Ye T , Yang Y , Liu G , Zhu L , SegMamba: long‐range sequential modeling mamba for 3D medical image segmentation. arXiv. Preprint posted online September 15, 2024. doi:10.48550/arXiv.2401.13560

[20] Pang S , Leung S , Nachum IB , Feng Q , Li S , Direct automated quantitative measurement of spine via cascade amplifier regression network. arXiv. Preprint posted online June 14, 2018. doi:10.48550/arXiv.1806.05570 10.1016/j.media.2019.04.012 31048199

[21] Li P , Zhou R , He J , Zhao S , Tian Y . A global‐frequency‐domain network for medical image segmentation. Comput Biol Med. 2023;164:107290. doi:10.1016/j.compbiomed.2023.107290 37579584 10.1016/j.compbiomed.2023.107290

[22] Wang W , Wang J , Chen C , et al. FreMIM: Fourier transform meets masked image modeling for medical image segmentation. In: 2024 IEEE/CVF Winter Conference on Applications of Computer Vision (WACV). IEEE; 2024:7845‐7855. doi:10.1109/WACV57701.2024.00768

[23] Chen Y , Zhang X , Peng L , He Y , Sun F , Sun H . Medical image segmentation network based on multi‐scale frequency domain filter. Neural Networks. 2024;175:106280. doi:10.1016/j.neunet.2024.106280 38579574 10.1016/j.neunet.2024.106280

[24] Xu Y , Quan R , Xu W , Huang Y , Chen X , Liu F . Advances in medical image segmentation: a comprehensive review of traditional, deep learning and hybrid approaches. Bioengineering. 2024;11(10):1034. doi:10.3390/bioengineering11101034 39451409 10.3390/bioengineering11101034 PMC11505408

[25] Tang S , Ran H , Yang S , et al. A frequency selection network for medical image segmentation. Heliyon. 2024;10(16):e35698. doi:10.1016/j.heliyon.2024.e35698 39220902 10.1016/j.heliyon.2024.e35698 PMC11365330

[26] Zhou Z , He A , Wu Y , Yao R , Xie X , Li T , Spatial‐frequency dual domain attention network for medical image segmentation. In: 2024 IEEE International Conference on Bioinformatics and Biomedicine (BIBM). IEEE; 2024:4076‐4081. doi:10.1109/BIBM62325.2024.10822613

[27] Meng D , Boyer E , Pujades S . Vertebrae localization, segmentation and identification using a graph optimization and an anatomic consistency cycle. Comput Med Imag Graph. 2023;107:102235. doi:10.1016/j.compmedimag.2023.102235 10.1016/j.compmedimag.2023.102235 37130486

[28] Saeed MU , Bin W , Sheng J , Ali G , Dastgir A . 3D MRU‐Net: a novel mobile residual U‐Net deep learning model for spine segmentation using computed tomography images. Biomed Signal Process Control. 2023;86:105153. doi:10.1016/j.bspc.2023.105153

[29] Pang S , Pang C , Zhao L , et al. SpineParseNet: spine parsing for volumetric MR image by a two‐stage segmentation framework with semantic image representation. IEEE Trans Med Imaging. 2021;40(1):262‐273. doi:10.1109/TMI.2020.3025087 32956047 10.1109/TMI.2020.3025087

[30] Peiris H , Hayat M , Chen Z , Egan G , Harandi M , A robust volumetric transformer for accurate 3D tumor segmentation. arXiv. Preprint posted online July 1, 2022. doi:10.48550/arXiv.2111.13300

[31] Zhou R , Feng Y , Wang G , et al. TSUBF‐net: trans‐spatial UNet‐like network with bi‐direction fusion for segmentation of adenoid hypertrophy in CT. arXiv. Preprint posted online December 1, 2024. doi:10.48550/arXiv.2412.00787

[32] Zhang Z , Liu T , Fan G , et al., SpineMamba: enhancing 3d spinal segmentation in clinical imaging through residual visual mamba layers and shape priors. Comput Med Imaging Graph. 2025;123:102531. doi:10.1016/j.compmedimag.2025.102531 40154009 10.1016/j.compmedimag.2025.102531

[33] Bozorgpour A , Azad B , Azad R , Velichko Y , Bagci U , Merhof D. HCA‐Net: Hierarchical Context Attention Network for Intervertebral Disc Semantic Labeling. arXiv. Preprint posted online November 21, 2023:arXiv:2311.12486. doi:10.48550/arXiv.2311.12486

